# Resistant starch and protein intake enhances fat oxidation and feelings of fullness in lean and overweight/obese women

**DOI:** 10.1186/s12937-015-0104-2

**Published:** 2015-10-29

**Authors:** Christopher L. Gentile, Emery Ward, Jens Juul Holst, Arne Astrup, Michael J. Ormsbee, Scott Connelly, Paul J. Arciero

**Affiliations:** 1 Human Nutrition and Metabolism Laboratory, Department of Health and Exercise Sciences, Skidmore College, 815 North Broadway, Saratoga Springs, NY 12866 USA; 2 Department of Food Science and Human Nutrition, Colorado State University, Fort Collins, CO USA; 3 Department of Biomedical Sciences, University of Copenhagen, Copenhagen, Denmark; 4 Department of Nutrition, Exercise and Sports, University of Copenhagen, Copenhagen, Denmark; 5 Department of Nutrition, Food and Exercise Sciences, Institute of Sports Sciences & Medicine, Florida State University, Tallahassee, FL USA; 6 Discipline of Biokinetics, Exercise & Leisure Studies, University of KwaZulu-Natal, Durban, South Africa; 7 Scott Connelly Foundation, Corona Del Mar, CA USA

**Keywords:** Resistant starch, High protein diets, Fat oxidation, Obesity, Satiety and hunger

## Abstract

**Background:**

Diets high in either resistant starch or protein have been shown to aid in weight management. We examined the effects of meals high in non-resistant or resistant starch with and without elevated protein intake on substrate utilization, energy expenditure, and satiety in lean and overweight/obese women.

**Methods:**

Women of varying levels of adiposity consumed one of four pancake test meals in a single-blind, randomized crossover design: 1) waxy maize (control) starch (WMS); 2) waxy maize starch and whey protein (WMS+WP); 3) resistant starch (RS); or 4) RS and whey protein (RS+WP).

**Results:**

Total post-prandial energy expenditure did not differ following any of the four test meals (WMS = 197.9 ± 8.9; WMS+WP = 188 ± 8.1; RS = 191.9 ± 8.9; RS+WP = 195.8 ± 8.7, kcals/180 min), although the combination of RS+WP, but not either intervention alone, significantly increased (*P* <0.01) fat oxidation (WMS = 89.5 ± 5.4; WMS+WP = 84.5 ± 7.2; RS = 97.4 ± 5.4; RS+WP = 107.8 ± 5.4, kcals/180 min). Measures of fullness increased (125 % vs. 45 %) and hunger decreased (55 % vs. 16 %) following WP supplemented versus non-whey conditions (WMS+WP, RS+WP vs. WMS, RS), whereas circulating hunger and satiety factors were not different among any of the test meals. However, peptide YY (PYY) was significantly elevated at 180 min following RS+WP meal.

**Conclusions:**

The combined consumption of dietary resistant starch and protein increases fat oxidation, PYY, and enhances feelings of satiety and fullness to levels that may be clinically relevant if maintained under chronic conditions. This trial was registered at clinicaltrials.gov as NCT02418429.

## Introduction

Dietary resistant starch (RS) has received considerable attention as a novel method to control body weight and prevent obesity [1]. Broadly categorized, resistant starch is any starch that passes undigested and unabsorbed through the small intestine to the colon [2]. Once in the colon, RS can be readily used as substrate for microbial fermentation, resulting in the production of short chain fatty acids and other metabolites that may have beneficial metabolic properties.

An increasing number of studies in animals [3, 4] and humans [5] support the suggestion that dietary RS may aid in weight control, and several mechanisms have been proposed to mediate this beneficial effect. First, given its resistance to digestion and absorption, replacing a portion of a meal with RS reduces the number of metabolizable calories [6]. Second, the production of short chain fatty acids as a result of RS fermentation may enhance total energy expenditure and/or fat oxidation [7]. Third, RS may reduce voluntary energy intake, in part by increasing the production of satiety signals such as peptide YY (PYY) and glucagon-like polypeptide −1 (GLP-1) [8].

There are four major types of RS (RS1-RS4), each of which contains specific chemical properties that render it resistant to digestion [9]. For example, RS1, found in whole grains and pasta with durum, contains a protein matrix which hinders its digestibility. RS2, found in common foods such as uncooked potatoes and unripe bananas, are resistant to carbohydrases until ripening or cooking. RS3 develops in starchy foods after storage because of the formation of double helices that render it resistant to enzymatic binding. RS4 is a chemically modified starch that resists enzymatic hydrolysis. To date, RS2 has received most of the attention for its beneficial metabolic properties [1]. However, RS4 was recently shown to reduce body weight more than RS2 in a murine model of obesity [10]. RS4 also increased resting energy expenditure and fat utilization compared to a waxy maize control in lean men after a single test meal [11]. Thus, RS4 represents an understudied form of RS that may beneficially affect human obesity and its comorbidities.

Increasing protein intake above commonly recommended levels is another dietary strategy that has been advocated for weight control and obesity prevention [12]. Similar to RS4, the effects of high protein diets are mediated, at least in part, through enhancing satiety and energy expenditure [13]. We recently demonstrated that increasing protein consumption to 35 % of total daily intake for eight weeks improved body composition and increased energy expenditure in obese adults [14].

The elevated energy expenditure and satiety elicited by diets high in either protein or RS4 likely occur via some, but not all, common mechanisms [15, 16]. Thus, it’s reasonable to speculate that diets high in both dietary protein and RS may have greater beneficial effects than either intervention alone. To begin to address this issue, we systematically compared the effects of meals high in non-RS constituents or RS4 with and without elevated protein intake on substrate utilization, energy expenditure, satiety and gastro-entero-pancreatic hormones in lean and overweight/obese women.

## Materials and methods

### Participants

A total of 70 women from the Saratoga Springs, NY area were recruited through newspaper advertisements and flyers and initially screened for participation, of whom 24 were eligible for participation. Participants were non-smoking, healthy women with no known cardiovascular or metabolic diseases as assessed by a medical history and examination by their personal physicians. Participants were middle-aged (45.8 ± 2.5 years), overweight/obese (BMI = 31.9 ± 1.4 kg/m^2^; % body fat = 41.2 ± 2.3) or lean (BMI = 21.0 ± 0.5 kg/m^2^; % body fat = 23.9 ± 1.4), and weight stable (±2 kg) for at least 6 months prior to beginning the study. Each participant provided informed written consent in adherence with the Skidmore College Human Subjects review board prior to participation, and the study was approved by the Human Subjects Institutional Review Board of Skidmore College. All experimental procedures were performed in accordance with the Federal Wide Assurance and related New York State regulations, which are consistent with the National Commission for the Protection of Human Subjects of Biomedical and Behavioral Research and in agreement with the Helsinki Declaration as revised in 1983. This trial was registered at clinicaltrials.gov as NCT02418429.

### Pancake test meal

On four separate visits to the Human Nutrition and Metabolism Laboratory, all subjects consumed one of four pancake test meals in a single-blind, randomized repeated measures crossover design: 1) waxy maize (control) starch (WMS, *n* = 16); 2) waxy maize starch and whey protein (WMS+WP, *n* = 9); 3) resistant starch (RS, *n* = 16); or 4) RS and whey protein (RS+WP, *n* = 16). Due to subject availability and time constraints, only 9 subjects (lean, *n* = 5; obese, *n* = 4) completed the WMS+WP test meal condition. Each pancake test meal was consumed together with water (180 ml) only. Pancakes were prepared following institutional guidelines using pre-gelatinized test starch, sugar, maltodextrin, vegetable oil, baking powder, egg, non-fat dry milk powder and water. All dry and wet ingredients were mixed together separately and then combined. Each meal consisted of three pancakes that were cooked on a non-stick griddle until golden brown. The nutritional composition of the four test meals are shown in Table 1.

**Table 1:** Nutritional analysis of pancake test meals

	WMS (Control)	WMS+WP	RS	RS+WP
Energy (kcal)	397	397	397	397
Waxy maize starch (g)	45	45	–	–
HDP from waxy maize starch (g)	–	–	40	40
Whey protein (g)	-	20.5	-	20.5
Sucrose (g)	4.5	4.5	4.5	4.5
Maltodextrin (g)	10.7	10.7	10.7	10.7
Milk powder (g)	21.1	0.9	21.1	0.9
Egg (g)	50	50	50	50
Baking powder (g)	4.8	4.8	4.8	4.8
Total carbohydrate (g)	73	60	73	60
Total fat (g)	5	5.5	5	5.5
Total protein (g)	15	26.8	15	26.8
Total fiber (g)	0	0	0	0

### Experimental design

**Fig. 1:**
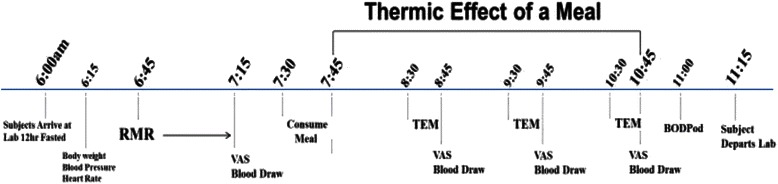
Test Day Timeline

The day before each of the four test days, participants were required to prepare their own meals and follow a standardized daily menu plan consisting of 25 % protein, 50 % carbohydrate, and 25 % fat, based on their estimated caloric needs. The final evening dinner meal the night prior to each test condition was consumed between 1800 and 2000 h and was identical for all four test conditions. All laboratory testing was conducted between 0600 and 0700 following a 12-hour fast (water was allowed) and at least a 24-hour restriction of physical activity, caffeine, and alcohol intake. Details regarding the test-day timeline are shown in Fig. 1. Briefly, upon arrival to the laboratory, body weight was measured (Befour Inc., model number FS0900) with participants wearing only shorts and a t-shirt. Following ~15 min of resting supine in a quiet, dimly lit room, resting metabolic rate (RMR) was measured for 30 min followed by a fasted blood draw (for plasma insulin, glucose, GIP, GLP-1, PYY, ghrelin, and leptin), and completion of visual analog scales (VAS) of hunger, desire to eat, and satiety (see testing procedures below). Subjects then consumed one of the four test meals (WMS; WMS+WP; RS; RS+WP) within 12 min. For 3 h following the completion of the test meals, subjects remained in a sedentary and supine position while serial blood samples were taken and VAS were completed (minutes 60, 120, 180). Indirect calorimetry was used to determine the thermic effect of the meal (TEM) (min 45–60, 105–120, 165–180). Following completion of the first test meal, participants were measured for body composition using the Life Measurements BODPod Body Composition Tracking System (Concord, CA).

### Resting metabolic rate (RMR) and Thermic Effect of a Meal (TEM)

RMR (kilocalories per minute) was measured using the ventilated hood technique [17] with a computerized open-circuit indirect calorimeter (Parvomedics, Truemax 2400, Salt Lake City, UT). Participants were not allowed to sleep and all measurements were obtained in the supine position following at least 15 min of quiet resting in a thermo-neutral (22–24 °C), semi-dark room. Following the RMR, a thermic effect of a meal (TEM) challenge was administered and postprandial thermogenesis was measured every 45 min for 180 min (TEM 45–60; 105–120; 165–180 min). Steady state was achieved for all participants during the final 10 min of each 15 min measurement period, and used in the calculation of TEM (minutes 0–5 were discarded). The total 180-minute TEM was calculated by taking an average of each 10-min TEM measurement and multiplying it by 60 min (0–60; 61–120; 121–180 min). Each of the three 60-min TEM periods was then summed for the 180-min TEM value.

The RQ and substrate utilization were also calculated from the gas exchange data using computer software from the calorimeter (Parvomedics, Truemax 2400) to provide fat and carbohydrate oxidation rates based on standardized caloric equivalents. The total 180-minute oxidation rates (fat and carbohydrate) were calculated using the exact same method described above for TEM. RMR (kcal/d) was calculated as the average of the test period. A 180 min TEM was chosen to capture the majority of the postprandial response. The total kilocalories eaten for each of the 4 test meals were isocaloric and only differed in the type of starch and protein content (Table 1). This study design allowed for the direct comparison of differences in starch composition and the macronutrient distribution of protein content on the thermogenic response. Test-retest intraclass correlation (r) and coefficient of variation (CV) in *n* = 14 is: RMR (Kcal/min) *r* = 0.92, 4.2 %, respectively.

### Plasma biomarkers

Venous blood samples (~20 ml) were obtained following the RMR and every hour after meal ingestion (TEM, minutes 60, 120, 180). Due to participant and resource constraints, no blood was obtained for WMS+WP. Blood was collected into EDTA-coated vacutainer tubes and centrifuged (Hettich Rotina 46R5) for 15 min at 2500 rpm at 4 °C. Plasma was then separated and stored at −70 °C in small aliquots until analyzed. Insulin, ghrelin, PYY, and GIP were determined using commercially available ELISA kits (Millipore, Inc. and DSL, Inc.). Plasma glucose concentrations were determined with a glucose analyzer using the glucose oxidase technique (GM7 Analyser, Analox Instruments, Lunenberg, MA). GLP-1 was analyzed using a radioimmunoassay with antiserum (no. 89390).

### Feelings of hunger, satiation and desire to eat

Visual analogue scales (VAS) were used to evaluate hunger, satiation, quantity of food that could be eaten, and desire to eat scores. Each VAS was 100 mm in length and anchored at each end. Participants were instructed to place a mark on the 100 mm line to indicate their levels of hunger, satiety, food quantity, and desire to eat. For hunger, a mark at 0 mm indicated no hunger, while a mark at 100 mm indicated extreme hunger. For satiety, a mark at 0 mm indicated no feeling of fullness, while a mark at 100 mm indicated an extreme feeling of fullness. For quantity of food that could be eaten, a mark at 0 mm indicated no food could be eaten, and a mark at 100 mm indicated a large quantity of food could be eaten. For desire to eat, a mark at 0 mm indicated no desire to eat, and a mark at 100 mm indicated extreme desire to eat. For each of the four measures (hunger, satiety, quantity of food eaten, and desire to eat), the degree to which each sensation was felt was quantified by measuring how far the mark was from the 0 mm point. For this measurement, a standard millimeter ruler was used and all scores were computed by the same investigator. VAS scales were completed during each of the four test meal conditions at baseline (RMR) and every hour during the TEM meal challenge (60, 120, and 180 min).

### Heart rate and blood pressure

Resting heart rate and blood pressure were obtained manually in the supine position as previously described [18]. Heart rate and blood pressure were obtained by a trained investigator on each of the four test meal days following the RMR measurement.

### Statistical analysis

**Fig. 2:**
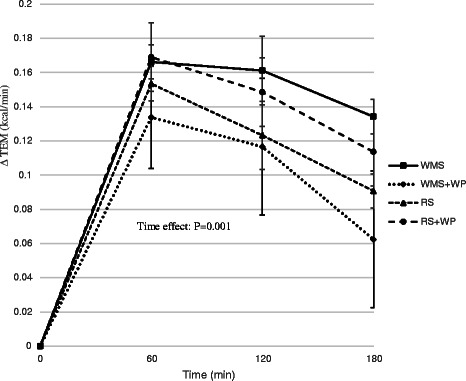
Effect of test meals on thermic response. Change in resting metabolic rate in the 180 min period immediately following the four test meals. *WMS* waxy maize control starch meal; *WMS+WP* waxy maize control starch and whey protein meal; *RS* resistant starch meal; *RS+WP* resistant starch and whey protein meal

**Fig. 3:**
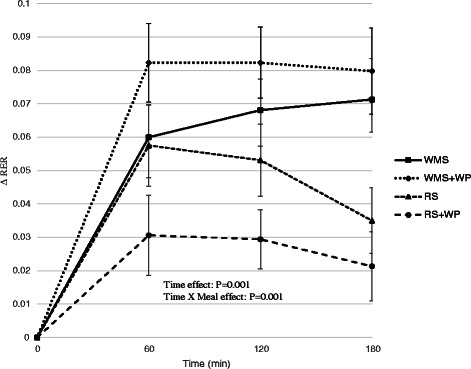
Effect of test meals on the respiratory exchange ratio. Change in respiratory exchange ratio in the 180 min period immediately following the four test meals. *WMS* waxy maize control starch meal; *WMS+WP* waxy maize control starch and whey protein meal; *RS* resistant starch meal; *RS+WP* resistant starch and whey protein meal

**Fig. 4:**
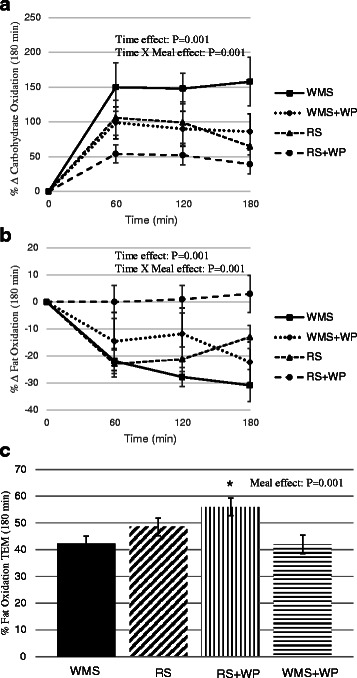
Effect of test meals on substrate oxidation. **a** Change (kcal/day) in carbohydrate oxidation in the 180 min period immediately following the four test meals; (**b**) Change (kcal/day) in fat oxidation in the 180 min period immediately following the four test meals; (**c**) Percent change in fat oxidation in the 180 min period immediately following the four test meals; *WMS* waxy maize control starch meal; *WMS+WP* waxy maize control starch and whey protein meal; *RS* resistant starch meal; *RS+WP* resistant starch and whey protein meal

**Fig. 5:**
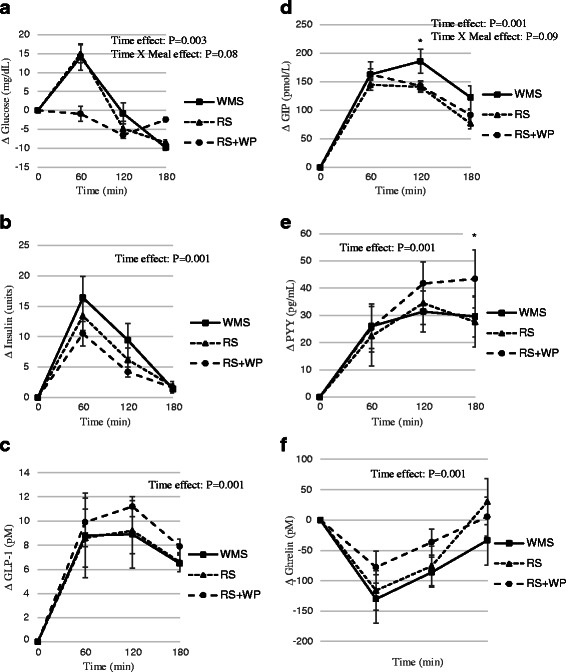
Effect of test meals on circulating factors. Change circulating factors in the 180 min period immediately following the four test meals. **a** glucose; (**b**) insulin; (**c**) glucagon like peptide-1; (**d**) gastric inhibitory polypeptide; (**e**) peptide YY 3–36; (**f**) ghrelin; *WMS* waxy maize control starch meal; *RS* resistant starch meal; *RS+WP* resistant starch and whey protein meal

**Fig. 6:**
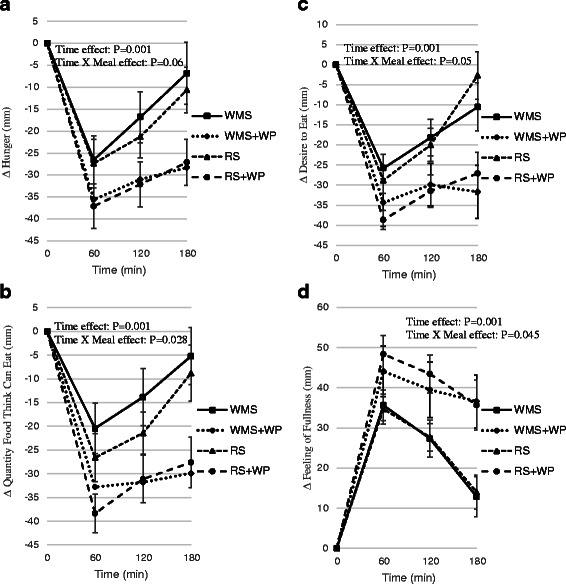
Effect of test meals on feelings of satiety, hunger, and desire to eat. Change in scores of hunger (**a**); quantity of food individuals though they could eat (**b**); feelings of fullness (**c**); and the desire to eat (**d**) in the 180 min period immediately following the four test meals; *WMS* waxy maize control starch meal; *WMS+WP* waxy maize control starch and whey protein meal; *RS* resistant starch meal; *RS+WP* resistant starch and whey protein mealTime effect: *P* = 0.001

Statistical analyses were performed using SPSS software (Ver. 21; IBM-SPSS Inc.). Significance was set at *p* <0.05. All values are reported as means ± SEM unless noted otherwise. Prior to the start of the study, subject number was determined from a power analysis based on the major outcome variables (TEM, fat oxidation, gastro-entero-pancreatic hormones, VAS) as reported by a previous study [10] with an alpha level set to 0.05 and a power of 0.8. This analysis determined we would require *n* = 12 participants to detect significant differences. Biomarker responses (glucose, insulin, GIP, GLP-1, ghrelin) were assessed by calculating the incremental area under the curve (AUC) using the trapezoid method. A 2 × 4 × 4 factor repeated measures ANOVA (2 groups; lean, obese: 4 test meals; WMS, WMS+WP, RS, RS+WP: 4 time points; 0, 60, 120, 180 min) was initially performed to determine differences among groups, test meals, and time points (time and group/test meal x time interactions). Thereafter, because no differences existed between lean and overweight/obese women for all variables, a 4 × 4 RMANOVA (meal × time) was run for all outcome variables. Absolute changes were calculated as the average baseline value (3 or 4 test meals) subtracted from each post-meal value (Figs. 2, 3, 5 and 6). Percent changes were calculated as the delta between baseline and each post meal time point divided by the baseline value (Fig. 4). Where significant main effects were identified, post hoc comparisons (paired sample *t*-test [time effects] and Tukey’s test [group differences]) were performed to locate differences. We utilized one-tailed *t*-tests for investigation of time effects for each test meal condition.

## Results

### Participants and compliance

Eight participants were not included in the data analysis due to scheduling conflicts (*n* = 5), dropout (*n* = 2), and noncompliance (*n* = 1). Baseline physical characteristics of the 16 subjects who completed testing are presented in Table 2 based on obesity status. As expected, obese women had significantly higher body weight, % body fat, BMI, and triglycerides, but lower HDL-Cholesterol levels compared to the lean women. Since no body composition-specific differences were observed in the thermogenic responses to the four test meals, lean and overweight/obese women were pooled together for all analyses.

**Table 2:** Subject characteristics

	Lean (*n* = 8)	Obese (*n* = 8)	Total Group (*n* = 16)
Age (years)	49.9 ± 2.1	41.6 ± 4.2	45.8 ± 2.5
Weight (kg)	55.9 ± 0.6	88.5 ± 2.2*	72.2 ± 2.7
Height (cm)	163.2 ± 2.1	167.0 ± 2.0	165.1 ± 1.4
BMI	21.0 ± 0.5	31.9 ± 1.4*	26.4 ± 1.5
Percent Fat (%)	23.9 ± 1.4	41.2 ± 2.3*	32.4 ± 2.6
Systolic BP (mmHg)	115.0 ± 1.3	115.5 ± 1.8	114.1 ± 1.2
Diastolic BP (mmHg)	73.1 ± 1.3	74.5 ± 1.6	74.5 ± 1.1
Resting HR (bpm)	56.0 ± 1.7	61.2 ± 1.3*	58.5 ± 1.3
Total Cholesterol (mg/dL)	200.4 ± 6.8	187.3 ± 13.8	195.1 ± 8.0
HDL (mg/dL)	88.3 ± 4.0	53.5 ± 4.6*	72.5 ± 5.5
LDL (mg/dL)	95.6 ± 6.9	105.1 ± 8.2	100.7 ± 21.0
Triglycerides (mg/dL)	54.8 ± 4.2	141.6 ± 27.0*	99.3 ± 18.5

Data presented as mean +/- SEM. ^*^, *P*<0.01

### Assessment of energy intake

Dietary intakes the day before each laboratory test meal were consistent among all conditions (data not shown). By design, total caloric intake reflected a caloric amount that did not include the energy cost of physical activity because participants were required to refrain from physical activity the day prior to each laboratory testing day. Additionally, the macronutrient intake provided a balance of carbohydrates (50 %), protein (25 %) and fat (25 %).

### Resting metabolic rate and thermic effect of a meal

Resting metabolic rate (RMR) was similar among all 4 test conditions for each participant. There was a main effect of time (*P* <0.01) during the TEM test meal (Fig. 2) but no time x meal interaction, suggesting that all test meals elicited similar thermogenic responses within the 3 h time period in which TEM data was collected. However, despite consuming identical calorie meals (~400 kcals), the respiratory quotient (RQ) was significantly reduced (*P* <0.01) following meals containing the resistance starch alone (RS) and with whey protein (RS+WP) (Fig. 3) with the greatest reduction occurring in the RS+WP meal (*P* <0.05). Similarly, the RS+WP test meal resulted in significantly heightened postprandial fat oxidation (WMS = 89.5 ± 5.4; WMS+WP = 84.5 ± 7.2; RS = 97.4 ± 5.4; RS+WP = 107.8 ± 5.4, kilocalories/180 min) and lower carbohydrate oxidation rates compared to isocaloric-matched test meals (Figs. 4a–c). It is important to note that postprandial thermogenesis remained higher at the end of our 180 min measurement period, suggesting an underestimation of the total TEM/RQ response.

### Plasma biomarkers

Plasma biomarkers are shown in Fig. 5a–f. Plasma glucose and insulin responses showed no main effects of time or time x meal interactions (Fig. 5a, b), however a trend existed for a time x meal interaction (*P* = 0.08) and the 60 min glucose was significantly (*P* <0.01) lower following RS+WP compared to RS and WMS. Plasma GLP-1, GIP, and PYY significantly (*P* <0.01) increased following meal ingestion (time effects) (Fig. 5c–e). There was a trend for a time x meal interaction for GIP (*P* = 0.09) and post hoc analysis showed plasma GIP was elevated (*P* <0.05) at 120 min in WMS compared to RS and RS+WP. Interestingly, PYY was elevated (*P* <0.05) at180 min in RS+WP compared to RS and WMS. Following meal ingestion, ghrelin decreased (*P* <0.05) following each meal with no time x meal interactions (Fig. 5f).

### Feelings of hunger, satiation and desire to eat

Feelings of hunger, satiation (amount of food that can be eaten), and desire to eat all decreased (*P* <0.01) following meal ingestion (time effects) (Fig. 6a–c), whereas fullness increased (Fig. 6d). Most strikingly, significant time x meal interactions (*P* <0.05) showed hunger, amount of food to be eaten, and desire to eat were lower and feelings of fullness were higher in meals containing the protein (RS+WP; WMS+WP) compared to WMS and RS. These findings imply that protein-rich meals (regardless of carbohydrate source) are associated with less feelings of hunger, amount of food that can be eaten, desire to eat and greater fullness compared with meals lower in protein.

## Discussion

The primary aim of the current study was to examine the effects of resistant starch (RS) alone and in combination with whey protein supplementation on energy expenditure, substrate utilization, and markers of hunger and satiety. We found that the combination of RS+WP, but not either intervention alone, significantly increased fat oxidation and PYY (at 180 min) post ingestion. Furthermore, measures of fullness and satiety were increased following whey protein supplementation, with no independent effect of RS.

There were no significant effects of resistant starch or whey protein on total postprandial energy expenditure. This is in accordance with the majority of previous studies that examined this issue [19–21]. However, Shimotoyodome et al., recently found that replacement of starch with RS4 in a mixed meal increased energy expenditure by approximately 70 % over a 3 h postprandial period [11]. The authors suggested that the discrepancy between their data and earlier studies was due to the lack of dietary fat as an energy substrate in the test meals of earlier studies. However, given that the test meals in the current study did contain dietary fat, other factors likely play a role. One possible explanation was the previous study included only lean men, whereas the current study included lean and obese women.

Perhaps the most interesting finding of the current study was that, compared to the control meal, fat oxidation was increased significantly when RS was combined with whey protein, but not when RS or whey protein was consumed alone. Recent studies have indicated that replacing digestible starch with RS (types two and four) in a test meal increases fat oxidation over a 3–6 h period [1, 11]. This effect may be due to production of short chain fatty acids following RS fermentation in the gut [22, 23], although the lack of these measurements in the ]current study precludes our ability to draw any firm conclusions. Still the marked effect of combining RS and whey protein on fat oxidation warrants further investigation.

Although we found a reduction in RQ in the RS test meal compared to control meal (*p* = 0.05), there was clearly an additional effect of combining RS with whey protein. These results confirm earlier findings that protein-rich meals (50 %), in particular those containing whey protein, increase fat oxidation compared to a low-protein (<5 %) meal [24]. It has been hypothesized that this effect may be elicited by lipolytic effects of glucagon. Although clear evidence for this mechanism is lacking and glucagon levels were not measured in the current study [24, 25], insulin was measured and was not statistically different among trials suggesting some other mechanism is responsible. There are obvious implications on body weight control if combined RS and whey protein consumption are determined to enhance fat oxidation; thus corroboration of these findings by future longer-term studies is important.

Test meals containing whey protein reduced feelings of hunger and increased feelings of fullness. These data are consistent with the well documented satiating effects of protein, and likely contribute to the beneficial effects of high protein diets on body weight [24, 26, 27]. RS alone had no effect on hunger and fullness, and the addition of RS to the whey protein meal elicited no further increases above protein alone. We also found no effects of the test meals on the hunger signal ghrelin (although a trend did exist, *P* = 0.09) and satiety signal GLP-1. In contrast, the satiety signal PYY was significantly elevated at 180 min in RS+WP compared to RS and WMS. Previous studies examining RS on subjective markers of hunger and satiety have generally found no acute effect in humans [5, 28], but numerous studies have found that RS increase gut-derived satiety signals in rodents [6, 8, 29]. Using pharmacologic and genetic inhibition, Zhou et al., [15] recently reported that PYY and GLP-1 play an important role in mediating the reduction in body fat following RS diets in rats. It’s unclear if these discrepant findings are due to intrinsic differences in research models, and more studies that examine alterations in circulating satiety/hunger signals in humans are necessary before any conclusions can be drawn.

We observed no significant differences in post prandial plasma glucose (although a trend existed, *P* = 0.08) or insulin concentrations in any of the test meals, which is in agreement with previous acute studies in humans that used a mixed meal [20, 21, 30]. Most studies that have reported a glucose lowering effect of RS provided test meals with varying amounts of fiber, protein or fat, which likely affected the glucose and insulin responses [31, 32]. For example, dietary protein is known to reduce the glycemic response in healthy and diabetic individuals [33]. Shimotoyodome et al., [11] recently demonstrated that a mixed meal containing RS4 significantly reduced postprandial glucose and insulin. The discrepant findings between that study and the current one are interesting given that test meals of both studies included pancakes and contained similar nutritional profiles, including the amount of RS4. The one notable difference is that men were examined in the previous report and women in the current study. However, there is no precedent or rationale for gender-specific differences in glucose responses following RS4 consumption; thus, the reason for the conflicting results is unclear.

Limitations of the current study should be noted. First, the whey protein used in the current study was a supplemental powder, and the physiological effects, including the effects on satiety and hunger, may differ between powder and whole food consumption. Second, as mentioned in the methods, due to participant constraints we were unable to determine plasma variables in the WMS+WP group, and thus the independent effects of whey protein on markers of satiety could not be examined.

## Conclusion

In conclusion, we found that a meal containing RS4 and whey protein significantly increased fat oxidation and PYY (at 180 min post-ingestion) in healthy women. The magnitude of change in fat oxidation is biologically relevant and could have important implications for body weight control if maintained under chronic conditions. Our data also lend further support to previous reports demonstrating that whey protein increases subjective satiety and reduces hunger. Future studies should examine the discrepancy between animal and human literature regarding the effects of RS on circulating satiety and hunger signals.

## Abbreviations

AUC: area under the curve
GLP-1: glucagon-like polypeptide −1
PYY: peptide YY
RMR: resting metabolic rate
RS: resistant starch
TEM: thermic effect of food
VAS: visual analog scales
WMS: waxy maize starch
WP: whey protein
